# Association Between Dietary Intake of One-Carbon Metabolism-Related Nutrients and Fluorosis in Guizhou, China

**DOI:** 10.3389/fnut.2021.700726

**Published:** 2021-09-28

**Authors:** Ting Chen, Na Tao, Sheng Yang, Dafang Cao, Xun Zhao, Donghong Wang, Jun Liu

**Affiliations:** ^1^Department of Preventive Medicine, School of Public Health, Zunyi Medical University, Zunyi, China; ^2^Department of Pharmacy, Affiliated Hospital of Zunyi Medical University, Zunyi, China; ^3^Department of Surveillance in Public Health, Center for Disease Control and Prevention of Renhuai City, Renhuai, China; ^4^Department of Chronic Diseases, Center for Diseases Control and Prevention of Zhijin County, Zhijin, China; ^5^Department of Gynaecology and Obstetrics, Affiliated Hospital of Zunyi Medical University, Zunyi, China

**Keywords:** one-carbon metabolism, fluorosis, dietary, betaine, choline, methionine, folate, cross-section study

## Abstract

**Objective:** This study aimed to investigate the associations between dietary one-carbon metabolism-related nutrients (betaine, choline, methionine, folate, vitamin B_6_, and vitamin B_12_) and fluorosis among the Chinese population in an area known for coal-burning fluorosis.

**Methods:** A cross-sectional study was conducted, with 653 fluorosis patients and 241 non-fluorosis participants. Dietary intake was acquired using a validated semi-quantitative 75-item food frequency questionnaire. The risk associations were assessed by unconditional logistical regression.

**Results:** We observed a significant inverse association between dietary betaine, total choline, methionine, folate, vitamin B_6_, and choline species and fluorosis. The adjusted *OR* (95% *CI*) in the highest quartile of consumption compared with the lowest were 0.59 (0.37–0.94) (*P*-trend = 0.010) for betaine intake, 0.45 (0.28–0.73) (*P-*trend = 0.001) for total choline intake, 0.45 (0.28–0.72) (*P*-trend < 0.001) for methionine intake, 0.39 (0.24–0.63) (*P*-trend < 0.001) for folate intake, 0.38 (0.24–0.62) (*P*-trend < 0.001) for vitamin B_6_ intake, and 0.46 (0.28–0.75) (*P*-trend = 0.001) for total choline plus betaine intake. Dietary intakes of choline-containing compounds, phosphatidylcholine, free choline, glycerophosphocholine, and phosphocholine were also inversely associated with lower fluorosis (all *P*-trend < 0.05). No significant associations were observed between dietary vitamin B_12_ or sphingomyelin and fluorosis.

**Conclusion:** The present study suggested that the higher dietary intakes of specific one-carbon metabolism-related nutrients, such as betaine, choline, methionine, folate, and vitamin B_6_, are associated with lower fluorosis prevalence.

## Introduction

Fluorine is an essential trace element, and the intake of an optimum concentration of fluoride anions is beneficial to the development of bones and teeth. However, excessive exposure to fluoride can lead to dental and skeletal fluorosis and damage non-skeletal systems (1). It has been estimated that over 70 million people may be affected by dental fluorosis worldwide, and the majority are located in Africa, India, China, the eastern Mediterranean, and southern Asia (2). The main type of fluorosis in China results from high levels of fluoride in drinking-water and pollution caused by burning fluoride-rich coal (coal-burning fluorosis). In total, 30.65 million people of China live in the coal-burning fluorosis risk areas, in 2017. Among them, there were 13.78 million who were found to suffer from dental fluorosis, and more than 1.7 million people suffering from skeletal fluorosis (3). Therefore, the prevention and control of fluorosis is an important public health problem in China.

Fluorosis is a complex disease and likely results from both oxidative stress and epigenetics (4–6). Recently, whole genome bisulfite sequencing (WGBS) indicated that the alterations in DNA methylation patterns (demethylation or hypermethylation) after fluoride exposure led to the abnormality of gene expression (6, 7). Additionally, exposure of fluoride was found to alter gene imprinting and methylation status of *H19, Peg3*, and *Line1* genes that may have a detrimental effect on developing a mouse embryo (8, 9). It has also been found that DNA methylation of *Cyclind-CDK4-p21* (10), neuronation (11), calcitonin-related polypeptide alpha (11), *BMP-2* (12), *BMP-7* (12) were associated with fluorosis. DNA methylation is determined by the availability of methyl groups from S-adenosylmethionine (SAM) (13), and is indirectly related to one-carbon metabolism nutrients, such as betaine, choline, methionine, folate, vitamin B_6_, and vitamin B_12_ (14). Animal studies found that choline and methionine attenuated fluoride toxicity (15, 16). These studies indicated that one-carbon metabolism nutrients may play a crucial role in preventing the fluorosis due to their involvement in the one-carbon metabolism pathway and their roles as methyl donors. However, only two small studies examined the association between parts of one-carbon nutrients and fluorosis. A cross-sectional study with 289 participants observed folate had a significant protective effect against fluorosis (17). Whereas, a case-control study with 45 cases and 41 controls reported that there was no significant difference between cases and controls in plasma levels of vitamin B_6_, vitamin B_12_, and folate (18). Thus, further studies are required to confirm the relationship due to the poverty of available evidence. Moreover, no epidemiological study explored the association of fluorosis with the dietary intake of betaine, choline, and methionine.

Therefore, the purpose of the present study was to comprehensively investigate the associations between dietary intake of six one-carbon metabolism nutrients (betaine, choline, methionine, folate, vitamin B_6_, and vitamin B_12_) and fluorosis among the Chinese population in areas known for coal-burning fluorosis. As alcohol can affect the DNA methylation by disrupting folate absorption (19), the study also evaluated whether the associations between one-carbon metabolism nutrients intake and fluorosis were modified by sex, alcohol drinking, smoking status, and urinary fluoride levels.

## Materials and Methods

### Ethics Statement

This study was approved by the Ethical Committee of Zunyi Medical University (No.2014-1-1003), Guizhou, China. All the participants in this study provided written informed consent before the interview.

### Study Participants

A population-based cross-sectional study was conducted between July and August, 2015 in a coal-burning area of Zhijin County Guizhou, China. A two–stage, clustered random sampling method was used in this study. Three towns (Chadian, Chengguan, and Puweng) were selected by a simple random sampling approach from the 10 towns in Zhijin County. Then, we randomly selected four villages from each selected town. In total, 12 villages (Dazai, Ganhe, Gaofeng, Guihua, Guohua, Hehua, Hualuo, Jiangyan, Moda, Shangzai, Yutang, and Xianfeng) were selected for the study. Participants who have lived in Zhijin for at least 10 years and aged 18–75 years were recruited through village doctors and the Center for Disease control and prevention (CDC) from randomly selected villages. The participants were excluded if they had a prior history of cancer, coronary heart disease, stroke, gout, or kidney disease, their dietary habits had manifestly changed during the previous 5 years, they were presently suffering from chronic diseases (cancer, diabetes, or hypertension, etc.) that might affect their dietary habits. In addition, the participants without complete questionnaire information were also excluded. Finally, a total of 894 (99.22% of these invited) participants out of 901 eligible participants were successfully interviewed. The urine samples of 718 subjects were obtained from the 894 participants interviewed.

### Diagnosis of Fluorosis

Participants diagnosed with either dental fluorosis or skeletal fluorosis were identified as fluorosis patients. The diagnoses of dental fluorosis (WS/T208-2011, China) and skeletal fluorosis (GB 16396-1996) were performed by a specialist from the CDC. A detailed description of the method is described in our previous studies (20, 21).

### Fluoride Concentration Measurements

The urine samples were collected and stored at −20°C before being determined. Urinary fluoride concentration was measured by the ion electrode method according to the standard WS/T 89-2015 (China). Further descriptions of the detection have previously been presented (20, 21). Briefly, the urine sample was taken out from the refrigerator at −20°C and thawed at room temperature. Further, 1 ml of each urine sample was taken, mixed with 24 ml of deionized water in a beaker. One fluoride Ionic Strength Adjustment (ISA) Powder Pillow (Hach, Cat: 258999, CO, USA) was added into these mixtures, stirred at a moderate rate. The concentration of urine fluoride in the mixture was then measured by HQ40d (Hach, CO, USA) portable meter, and the concentration of fluorine in the urine of the subjects was calculated according to the corresponding proportion. According to WS/T 256-2005 requirements, a normal urinary fluoride concentration is 1.6 mg/L or less (22). The coefficient of variation for the urinary fluoride was 2.29%.

### Assessment of Dietary One-Carbon Metabolism-Related Nutrients Intake

Dietary consumption information was collected from all the participants through the medium of a semi-quantitative 75-item food frequency questionnaire (FFQ). The validity and reproducibility of the FFQ were assessed in the previous studies (13, 23). The consultation period for the dietary intake investigation was the year prior to the interview of all participants. Participants selected from five qualitative responses for each item on the FFQ, and reported dietary intake frequency ranging from never, per year, per month, and per week to per day to evaluate the intake amount. The pictures of food portion sizes were offered to help the participants to estimate the quantity of food consumption. Each food item was converted into daily consumption (grams per day). Average daily dietary intakes of one-carbon metabolism-related nutrients were estimated by multiplying the consumption frequency and portion size of each food item by the nutrient content, referring to the Chinese food composition database (24).

Intakes of B_6_, B_12_, folate, and methionine were calculated on the basis of the Chinese food composition database (24). Due to the limited Chinese food composition data available, the intake of betaine, choline, and choline subclasses, such as choline from free choline, glycerophosphocholine, phosphocholine, phosphatidylcholine, and sphingomyelin, were calculated using US Department of Agriculture database (USDA) (25). The sum of intakes of the five subclasses was estimated as total choline intake. The previous studies have shown that the correlations were high between intakes of common nutrients derived from the USDA and Chinese databases (e.g., r > 0.90 for B vitamins and methionine), indicating the validity of using the USDA database in our Chinese population to estimate the choline and betaine intakes (26).

### Data Collection

Face-to-face interviews were performed to obtain the following detail information: sociodemographic characteristics (e.g., age, gender, marital status, level of education, household income, and other factors), lifestyle habits (e.g., intake of tobacco, tea drinking, and alcohol consumption), history of chronic diseases (e.g., cancer, hypertension, diabetes, heart disease, gout or renal diseases, and stroke), and history of nutritional supplements (e.g., calcium and multivitamins). Those participants with improved stove or other fuel type related situations were reported. In the present study, the participants who smoked at least five packs of cigarettes a year were defined as regular smokers. In addition, those who were passively exposed to the tobacco smoking of others for at least 5 min/day consecutively were passive smokers. An alcohol drinker was defined as someone drinking alcohol at least once a week for more than 6 months subsequently. Tea drinkers were defined as someone drinking tea at least two times per week in the past year.

### Statistics Analysis

Data were analyzed using SPSS 18.0 software (SPSS Inc., IL, USA). All *P*-values were two tailed, and below 0.05 were considered statistically significant. Continuous data were expressed as mean (M) and SD (s) or median (P_25_, P_75_), while the categorical variables were expressed as proportions. A regression residual nutrient method illustrated by Willett et al. (27) was used to adjust the energy intake of betaine, total choline, methionine, folate, vitamins B_6_ and B_12_, and choline subclasses. The analyses of two independent sample *t*-test were used to analyze the mean differences between the characteristics of the participants (normal distribution). The *Mann–Whitney* test was used to compare the median consumption levels between the fluorosis and non-fluorosis participants (skewed distribution), and χ^2^-tests were used to compare differences in the frequencies of categorical variables. We categorized energy-adjusted dietary intake of betaine, total choline, methionine, folate, vitamins B_6_ and B_12_, and choline subclasses into quartiles (Q1–Q4). The lowest quartile (Q1) served as the reference group. An unconditional logistic regression model was used to evaluate the associations between energy-adjusted dietary one-carbon metabolism nutrient intakes and endemic fluorosis, and to calculate the odds ratios (*ORs*) with 95% *CIs*. The multivariate models were used to adjust the potential confounding factors, such as age (continuous), sex (men/women), marital status (married or cohabitated/divorces or widowed/unmarried), education level (illiteracy/primary school/secondary school/high school or above), household income ( ≤ 1000/1001–2000/2001–4000/4001–6000/≥ 6001), smoker (yes/no), passive smoking exposure (yes/no), tea drinker (yes/no), alcohol drinker (yes/no), use of coal to roast grains or chili (yes/no), washing dry grains or chili before use (yes/no), fuel type (raw coal/mixed coal/fire wood/others), and improved stove use (yes/no) (Model 1). To further evaluate whether the associations were modified by fluorosis-related behavior and nutrients, subsequent models were adjusted for dietary factors, such as roasted chili consumption (continuous), dietary calcium intake (continuous), total energy intake, roasted corn intake (continuous), and dietary fiber intake (continuous) (Model 2). The tests for trend were conducted by entering the ordinal values of the quartile of dietary one-carbon metabolism-related nutrients as continuous variables in the models.

The stratified analyses according to sex (men/women), alcohol drinker (yes/no), smoker (yes/no), and urinary fluoride levels ( ≤ 1.6 vs. > 1.6 mg/L) were also conducted to explore whether the folate-fluorosis associations were modified. The *P* for interactions between potential factors and folate were calculated from the multivariate logistic regression models by multiplicative interaction items. The subgroup analyses for dental fluorosis or skeletal fluorosis were performed for the relationships between dietary one-carbon metabolism nutrients and fluorosis. Furthermore, the sensitivity of results excluding participants with multi-vitamin supplements was evaluated.

## Results

Of the 894 participants (412 men and 482 women), 653 were diagnosed to have endemic fluorosis (311 men and 342 women), and 241 did not have fluorosis (101 men and 140 women). Table 1 shows the baseline demographic characteristics of study topics and selected risk factors of endemic fluorosis. The mean (±s) age of fluorosis group was 50.4 (±12.3) years, and the non-fluorosis was 47.0 (±14.6) years. Compared with the non-fluorosis subjects, the fluorosis patients tended to have lower education level and household annual income, be older and less likely to use an improved stove, more likely use coal to roast grains and chilis, and use mixed coal fuel types. The fluorosis patients had lower intakes of betaine, total choline, methionine, folate, vitamins B_6_, and higher levels of urinary fluoride compared with the non-fluorosis participants. In addition, compared with the fluorosis patients, the non-fluorosis participants tended to have a higher intake of the main choline-containing compounds, except for the choline from sphingomyelin (Table 2).

**Table 1:** Socio-demographic characteristics and selected risk factors of the participants in coal-burning fluorosis area in Guizhou.

**Characteristics**	**Fluorosis group (*n* = 653)**	**Non-flluorosis group (*n* = 241)**	***P*-value**^†^****
Age, year (mean ± s)	50.4 ± 12.3	47.0 ±14.6	0.001
Sex, *n* (%)			0.128
Men	311 (47.6)	101 (41.9)	
Women	342 (52.4)	140 (58.1)	
Marital status, *n* (%)			0.071
Married or cohabitated	554 (84.8)	199 (82.6)	
Divorces or widowed	72 (11.0)	23 (9.5)	
Unmarried	27 (4.1)	19 (7.9)	
Income (yuan/capita/year), *n* (%)			0.022
≤ 1,000	141 (21.6)	36 (14.9)	
1,001–2,000	186 (28.5)	69 (28.6)	
2,001–4,000	177 (27.1)	63 (26.1)	
4,001–6,000	84 (12.9)	32 (13.3)	
≥ 6,001	65 (10.0)	41 (17.0)	
Educational level, *n* (%)			< 0.001
Illiteracy	340 (52.1)	83 (34.4)	
Primary school	224 (34.3)	75 (31.1)	
Secondary school	75 (11.5)	55 (22.8)	
High school or above	14 (2.1)	28 (11.6)	
Fuel type, *n* (%)			< 0.001
Raw coal	395 (60.5)	137 (56.8)	
Mixed coal	152 (23.3)	40 (16.6)	
Firewood	15 (2.3)	17 (7.1)	
Others	91 (13.9)	47 (19.5)	
Smoker ^§^, *n* (%)	277 (42.4)	85 (35.3)	0.053
Passive smoker^‡^, n (%)	429 (65.7)	163 (67.6)	0.587
Alcohol drinker^‡^, *n* (%)	202 (30.9)	73 (30.3)	0.853
Tea drinker^⁑^, *n* (%)	249 (38.1)	90 (37.3)	0.830
Using an improved stove^||^, *n* (%)	465 (71.2)	209 (86.7)	< 0.001
Using coal to roast grains and chilis^|^, *n* (%)	391 (59.9)	114 (47.3)	0.001
Washing dry grains and chilis before use, *n* (%)	611 (93.6)	233 (96.7)	0.072

† *Between fluorosis and non-fluorosis differences on categorical variables were analyzed with chi-square tests, and on continuous variables were with two independent sample t-tests, incasing of skewed distributions, non-parametric t-test were used. s, SD. n (%), number of cases (proportion)*.

§ *Smokers were defined as smoking at least five packs of cigarettes per year*.

‡ *Passive smokers were defined as having passive exposed to tobacco smoking at least consecutive 5 min daily*.

‡ *Alcohol drinkers were defined as having beer, white wine, or red wine at least once weekly over the past year*.

⁑ *Tea drinkers were identified as having tea at the least two times per week*.

|| *Improve open oven to reduce fluoride pollution indoors by excluding fluoride out of room*.

| *Burning fluoride-rich coal for roasted grain and chili*.

**Table 2:** Comparison of energy, urinary fluoride, folate, methionine, vitamins B_6_ and B_12_, total choline, betaine, and individual choline-containing compounds intake^‡^ between fluorosis and non-fluorosis.

**Food items**	**Fluorosis group (*****n*** **=** **653)**			**Non- fluorosis group (*****n*** **=** **241)**			***P*- value^‡^**
	**Mean**	**SD**	**Median (25th, 75th)**	**Mean**	**SD**	**Median (25th, 75th)**	
Energy (kcal/day)	2737.34	988.11	2611.72 (1991.63, 3402.92)	2693.08	945.77	2552.13 (2008.77, 3219.32)	0.548
Urinary fluoride (mg/L)	1.68	0.99	1.42 (1.03, 2.08)	1.41	1.20	1.17 (0.81, 1.67)	< 0.001
Folate (μg/day)	445.84	172.54	414.70 (321.82, 528.28)	495.87	179.46	478.66 (362.81, 605.91)	< 0.001
Methionine (mg/day)	2098.42	757.56	2019.83 (1588.28, 2505.07)	2299.76	774.86	2282.60 (1713.16, 2814.31)	< 0.001
Vitamin B_6_ (mg/day)	1.56	0.58	1.48 (1.14, 1.90)	1.78	0.63	1.77 (1.35, 2.16)	< 0.001
Vitamin B_12_ (μg/day)	2.25	1.59	1.95 (1.06, 3.05)	2.46	1.64	2.21 (1.13, 3.36)	0.063
^†^Total choline (mg/day)	212.24	128.86	180.89 (125.72, 264.05)	244.78	129.63	214.80 (149.63, 316.90)	< 0.001
Phosphatidylcholine (mg/day)	122.76	96.95	98.22 (59.12, 152.86)	139.45	93.56	115.72 (74.18, 192.26)	0.001
Free choline (mg/day)	53.35	27.05	46.99 (34.76, 64.98)	64.49	32.64	55.62 (40.21, 81.21)	< 0.001
Glycerophosphocholine (mg/day)	20.71	9.95	18.78 (14.55, 24.47)	22.21	9.85	19.90 (15.61, 27.41)	0.016
Phosphocholine (mg/day)	9.71	5.55	8.43 (5.71, 12.47)	12.65	7.82	10.40 (7.73, 15.78)	< 0.001
Sphingomyelin (mg/day)	6.09	5.64	4.60 (2.27, 8.05)	6.46	5.82	4.99 (2.54, 8.50)	0.227
Betaine (mg/day)	133.81	144.05	91.77 (54.67, 149.30)	191.46	291.16	110.25 (68.17, 199.36)	0.001
Total choline +betaine (mg/day)	346.05	216.07	291.85 (201.85, 423.78)	436.25	351.79	361.40 (241.61, 530.55)	< 0.001
Ca (mg/day)	520.20	293.41	451.52 (314.77, 656.26)	558.08	314.74	474.85 (335.25, 710.40)	0.105
Roasted chili (g/day)	13.21	17.62	5.79 (2.41, 12.78)	12.55	16.90	6.03 (2.41, 13.50)	0.780
Roasted grain (g/day)	32.92	66.61	13.70 (4.11, 32.88)	24.19	32.70	13.70 (4.73, 27.95)	0.719

*^⁑^Intake of folate, methionine, vitamins B_6_ and B_12_, total choline, individual choline-containing compounds, and betaine were adjusted for the daily energy intake by the means of the residual method. P_25_ and P_75_ represent the 25th and 75th percentile. SD*.

‡ *Mann-Whitney test was used to compare the median consumption levels between fluorosis and non-fluorosis*.

† *Total choline intake was calculated by summing of phosphatidylcholine, free choline, glycerophosphocholine, phosphocholine, and sphingomyelin*.

Table 3 presents the *ORs* and 95% *CIs* for fluorosis according to quartiles of one-carbon metabolism-related nutrients intakes. After adjustment by socio-demographic and lifestyle factors, the *ORs* for fluorosis occurrence in the highest quartile, compared with the lowest quartile of uptakes, were 0.63 (95% *CI*: 0.40–0.99) (*P*-trend = 0.020) for betaine, 0.48 (95% *CI*: 0.30–0.78) (*P*-trend = 0.003) for total choline, 0.48 (95% *CI*: 0.30–0.77) (*P*-trend < 0.001) for methionine, 0.45 (95% *CI*: 0.28–0.73) (*P*-trend < 0.001) for folate, 0.47 (95% *CI*: 0.30–0.74) (*P*-trend < 0.001) for vitamin B_6_, and 0.80 (95% *CI*: 0.51–1.25) (*P*-trend = 0.164) for vitamin B_12_. As choline is irreversibly converted into betaine in its role as a methyl donor, we also assessed the combined dietary of choline plus betaine and showed an inverse association with fluorosis prevalence. The adjusted *OR* (95% *CI*) was 0.51 (0.32–0.81) (*P*-trend = 0.005) for the fourth quartile compared with the first quartile intake. The inverse association did not change after further adjustment for dietary factors, such as calcium intake, total calorie intake, roasted chili and grain consumption, and dietary fiber intake. There was no evidence for interaction on fluorosis occurrence from folate with other one-carbon metabolism-related nutrients betaine, total choline, methionine, and vitamin B_6_ (*P* for interactions were 0.607, 0.657, 0.658, and 0.745, respectively).

**Table 3:** Odds ratios (*ORs*) and 95% *CIs* of fluorosis for quartiles of dietary energy-adjusted one-carbon metabolism-related nutrients intakes.

**Items**	**Quaritle of intake**				***P*-trend**
	**Q1**	**Q2**	**Q3**	**Q4**	
Betaine (mg/day)	29.43	77.35	122.91	261.21	
No. Cases/Controls	173/50	174/50	162/62	144/79	
Crude OR (95% CI)	1	1.00 (0.65, 1.57)	0.76 (0.49, 1.16)	0.53 (0.35, 0.80)^**^	0.001
Model 1^†^	1	1.01 (0.63, 1.63)	0.73 (0.46, 1.16)	0.63 (0.40, 0.99)^*^	0.020
Model 2^‡^	1	1.03 (0.64, 1.65)	0.72 (0.45, 1.14)	0.59 (0.37, 0.94)^*^	0.010
Total choline (mg/day)	101.35	159.75	225.20	372.46	
No. Cases /Controls	180/43	168/56	160/64	145/78	
Crude OR (95% CI)	1	0.72 (0.46, 1.12)	0.60 (0.38, 0.93)^*^	0.44 (0.29, 0.68)^**^	< 0.001
Model 1^†^	1	0.66 (0.41, 1.06)	0.59 (0.37, 0.94)^*^	0.48 (0.30, 0.78)^**^	0.003
Model 2^‡^	1	0.63 (0.39, 1.02)	0.57 (0.35, 0.91)^*^	0.45 (0.28, 0.73)^**^	0.001
Methionine (mg/day)	1298.85	1847.27	2319.86	3030.74	
No. cases /Controls	171/52	180/44	164/60	138/85	
Crude OR (95% CI)	1	1.24 (0.79, 1.96)	0.83 (0.54, 1.28)	0.49 (0.33, 0.75)^**^	< 0.001
Model 1^†^	1	1.16 (0.72, 1.88)	0.78 (0.49, 1.25)	0.48 (0.30, 0.77)^**^	< 0.001
Model 2^‡^	1	1.14 (0.70, 1.86)	0.74 (0.46, 1.20)	0.45 (0.28, 0.72)^**^	< 0.001
Folate (μg/day)	269.73	382.34	486.82	666.75	
No. Cases /Controls	179/44	177/47	153/71	144/79	
Crude OR (95% CI)	1	0.93 (0.58, 1.47)	0.53 (0.34, 0.82)^**^	0.45 (0.29, 0.69)^**^	< 0.001
Model 1^†^	1	0.95 (0.58, 1.54)	0.50 (0.31, 0.80)^**^	0.45 (0.28, 0.73)^**^	< 0.001
Model 2^‡^	1	0.88 (0.54, 1.44)	0.45 (0.28, 0.72)^**^	0.39 (0.24, 0.63)^**^	< 0.001
Vitamin B_6_ (mg/day)	0.96	1.37	1.76	2.35	
No. Cases /Controls	177/46	180/44	153/71	143/80	
Crude OR (95% CI)	1	1.06 (0.67, 1.69)	0.56 (0.36, 0.86)^**^	0.47 (0.30, 0.71)^**^	< 0.001
Model 1^†^	1	1.04 (0.64, 1.70)	0.53 (0.33, 0.84)^**^	0.47 (0.30, 0.74)^**^	< 0.001
Model 2^‡^	1	0.97 (0.59, 1.59)	0.48 (0.30, 0.78)^**^	0.38 (0.24, 0.62)^**^	< 0.001
Vitamin B_12_ (μg/day)	0.68	1.55	2.52	4.18	
No. Cases /Controls	168/55	172/52	160/64	153/70	
Crude OR (95% CI)	1	1.08 (0.70, 1.67)	0.82 (0.54, 1.25)	0.72 (0.47, 1.08)	0.055
Model 1^†^	1	1.17 (0.74, 1.85)	0.84 (0.53, 1.32)	0.80 (0.51, 1.25)	0.164
Model 2^‡^	1	1.16 (0.72, 1.84)	0.88 (0.56, 1.39)	0.81 (0.51, 1.28)	0.217
Total choline + betaine	154.98	260.43	367.34	611.63	
No. Cases /Controls	181/42	169/55	161/63	142/81	
Crude OR (95% CI)	1	0.71 (0.45, 1.12)	0.59 (0.38, 0.93)^*^	0.41 (0.26, 0.63)^**^	< 0.001
Model 1^†^	1	0.66 (0.41, 1.07)	0.59 (0.36, 0.94)^*^	0.51 (0.32, 0.81)^**^	0.005
Model 2^‡^	1	0.66 (0.41, 1.07)	0.55 (0.34, 0.89)^*^	0.46 (0.28, 0.75)^**^	0.001

*Crude OR (95% CI) without further adjustment*.

† *OR was adjusted for covariates, such as age, sex, marital status, education level, income, smoking status, passive smoking status, alcohol drinking status, tea drinking status, using coal to roast grains or chili, washing dry grains or chili before use, fuel type, and using improved stove*.

‡ *OR was adjusted for the various above confounders and calcium intake, roasted chili and grains consumption, total energy intake, and dietary fiber intake*.

* *p < 0.05*,

** *p < 0.01*.

For the five main choline-containing compounds, the higher dietary intakes of choline from phosphatidylcholine, free choline, choline from glycerophosphocholine, and choline from phosphocholine were associated with lower fluorosis occurrence. After adjustment for various dietary and non-dietary factors, highest vs. lowest quartile *ORs* for compounds uptake were 0.45 (95% *CI*: 0.28–0.72) (*P-*trend = 0.002) for phosphatidylcholine, 0.32 (95% *CI*: 0.19–0.52) (*P*-trend < 0.001) for free choline, 0.52 (95% *CI*: 0.32–0.84) (*P*-trend = 0.015) for glycerophosphocholine, and 0.35 (95% *CI*: 0.21–0.57) (*P*-trend < 0.001) for phosphocholine. In contrast, no statistical association was observed between dietary sphingomyelin and fluorosis (*P*-trend = 0.666) (Table 4).

**Table 4:** *ORs* and 95% *CIs* of fluorosis according to quartiles of dietary energy-adjusted five main choline-containing compounds intakes.

**Items**	**Quaritle of intake**				***P*-trend**
	**Q1**	**Q2**	**Q3**	**Q4**	
Phosphatidylcholine (mg/day)	44.65	82.13	125.23	223.26	
No. cases/Controls	182/41	164/60	162/62	145/78	
Crude OR (95% CI)	1	0.62 (0.39, 0.97)^*^	0.59 (0.38, 0.92) ^*^	0.42 (0.27, 0.65)^**^	< 0.001
Model 1^†^	1	0.61 (0.38, 0.98)^*^	0.59 (0.36, 0.94)^*^	0.46 (0.29, 0.75)^**^	0.002
Model 2^‡^	1	0.60 (0.37, 0.97)^*^	0.58 (0.36, 0.93)^*^	0.45 (0.28, 0.72)^**^	0.002
Free choline (mg/day)	29.48	42.51	57.16	91.30	
No. cases /Controls	184/39	171/53	160/64	138/85	
Crude OR (95% CI)	1	0.68 (0.43, 1.09)	0.53 (0.34, 0.83)^**^	0.34 (0.22, 0.53)^**^	< 0.001
Model 1^†^	1	0.64 (0.39, 1.04)	0.56 (0.35, 0.91)^*^	0.36 (0.23, 0.59)^**^	< 0.001
Model 2^‡^	1	0.63 (0.39, 1.03)	0.52 (0.32, 0.85)^**^	0.32 (0.19, 0.52)^**^	< 0.001
Glycerophosphocholine (mg/day)	11.29	16.87	21.88	31.39	
No. cases/Controls	177/46	162/62	160/64	154/69	
Crude OR (95% CI)	1	0.68 (0.44, 1.05)	0.65 (0.42, 1.00)	0.58 (0.38, 0.89) ^*^	0.017
Model 1^†^	1	0.57 (0.36, 0.91)^*^	0.57 (0.36, 0.92)^*^	0.56 (0.35, 0.90)^*^	0.025
Model 2^‡^	1	0.53 (0.33, 0.86)^*^	0.54 (0.34, 0.87)^*^	0.52 (0.32, 0.84)^**^	0.015
Phosphocholine (mg/day)	4.32	7.51	10.83	17.78	
No. Cases/Controls	185/38	173/51	152/72	143/80	
Crude OR (95% CI)	1	0.70 (0.44, 1.11)	0.43 (0.28, 0.68)^**^	0.37 (0.24, 0.57)^**^	< 0.001
Model 1^†^	1	0.72 (0.44, 1.17)	0.44 (0.27, 0.71)^**^	0.40 (0.25, 0.65)^**^	< 0.001
Model 2^‡^	1	0.67 (0.40, 1.09)	0.40 (0.25, 0.65)^**^	0.35 (0.21, 0.57)^**^	< 0.001
Sphingomyelin (mg/day)	1.31	3.53	6.33	11.76	
No. cases/Controls	167/54	160/61	162/60	158/63	
Crude OR (95% CI)	1	0.87 (0.57, 1.34)	0.84 (0.55, 1.28)	0.78 (0.51, 1.18)	0.238
Model 1^†^	1	0.95 (0.60, 1.50)	0.87 (0.56, 1.37)	0.92 (0.58,1.46)	0.653
Model 2^‡^	1	0.98 (0.62, 1.56)	0.86 (0.54, 1.36)	0.94 (0.59, 1.49)	0.666

*Crude OR (95% CI) without further adjustment*.

† *See Table 3 for the covariates*.

‡ *See Table 3 for the covariates*.

* *p < 0.05*,

** *p < 0.01*.

The stratified analyses of alcohol drinking status and urinary fluoride levels showed an inverse association between folate intake and fluorosis only among the participants without alcohol consumption (*OR* for the Q4 vs. Q1 was 0.30 [95% *CI*: 0.17–0.56, *P*-trend < 0.001]), and among those with low urinary fluoride levels (*OR* for the Q4 vs. Q1 was 0.31 [95% *CI*: 0.15–0.61, *P*-trend < 0.001]), but not among those with alcohol consumption (*OR* for the Q4 vs. Q1 was 0.65 [95% CI: 0.25–1.62, *P*-trend = 0.056]), and those with high urinary fluoride levels (*OR* for the Q4 vs. Q1 was 0.63 [95% *CI*: 0.21–1.84, *P*-trend = 0.237]). However, the interaction effect between folate intake and alcohol consumption or urinary fluoride level was not statistically significant (*P* for interactions were 0.583 and 0.575). Similar inverse associations were found after stratified by sex and smoking status (Supplementary Table 1). The analyses stratified by the above factors were also conducted for other one-carbon metabolism nutrients. However, no significant interactions were observed (*P* for interactions ranged from 0.094 to 0.929, Supplementary Tables 2–5).

Subgroup of analyses revealed that the inverse associations between one-carbon metabolism-related nutrients (betaine, choline, methionine, folate, and vitamin B_6_) and fluorosis occurrence were found in both dental fluorosis and skeletal fluorosis (Table 5). Additionally, sensitivity analysis by excluding those participants who reported use of multi-vitamins supplements (five for cases and eight for controls) showed no substantial change (data not shown).

**Table 5:** *ORs* and 95% *CIs* of fluorosis according to quartiles of dietary energy-adjusted one-carbon metabolism-related nutrients by fluorosis damage sites.

**Dietary variables**	**Dental fluorosis (*****n*** **=** **870)**			**Skeletal fluorosis (*****n*** **=** **561)**		
	**Case/control**	**OR^‡^ (95% CI)**	***P*-trend**	**Case/control**	**OR^‡^ (95% CI)**	***P*-trend**
Betaine (mg/day)			0.015			0.009
Q1	168/49	1		93/47	1	
Q2	164/54	0.89 (0.56, 1.44)		88/52	1.01 (0.58, 1.76)	
Q3	158/60	0.72 (0.45, 1.16)		77/64	0.63 (0.37, 1.10)	
Q4	139/78	0.58 (0.36, 0.94)^*^		62/78	0.54 (0.30, 0.95)^*^	
Total choline (mg/day)			0.005			0.001
Q1	172/45	1		96/44		
Q2	162/56	0.67 (0.42, 1.08)		89/51	0.91 (0.52, 1.59)	
Q3	154/64	0.60 (0.37, 0.97)^*^		75/66	0.62 (0.35, 1.08)	
Q4	141/76	0.50 (0.31, 0.80)^**^		60/80	0.42 (0.24, 0.74)^**^	
Methionine (mg/day)			0.001			< 0.001
Q1	163/54	1		92/48	1	
Q2	175/43	1.25 (0.77, 2.04)		93/47	1.38 (0.77, 2.47)	
Q3	157/61	0.77 (0.48, 1.23)		82/59	0.74 (0.42, 1.33)	
Q4	134/83	0.49 (0.31, 0.79)^**^		53/87	0.35 (0.20, 0.64)^**^	
Folate (μg/day)			< 0.001			< 0.001
Q1	172/45	1		97/43	1	
Q2	171/47	0.93 (0.57, 1.53)		91/49	0.97 (0.55, 1.72)	
Q3	147/71	0.47 (0.29, 0.76)^**^		71/70	0.41 (0.23, 0.74)^**^	
Q4	139/78	0.42 (0.25, 0.68)^**^		61/79	0.37 (0.21, 0.68)^**^	
Vitamin B_6_ (mg/day)			< 0.001			< 0.001
Q1	170/47	1		94/46	1	
Q2	174/44	1.02 (0.62, 1.67)		94/46	1.03 (0.58, 1.82)	
Q3	147/71	0.50 (0.31, 0.81)^**^		72/69	0.45 (0.26, 0.80)^**^	
Q4	138/79	0.40 (0.25, 0.66)^**^		60/80	0.36 (0.20, 0.65)^**^	
Vitamin B_12_ (mg/day)			0.322			0.114
Q1	165/53	1		90/50	1	
Q2	155/63	1.16 (0.73, 1.84)		85/55	1.09 (0.62, 1.89)	
Q3	155/63	0.94 (0.59, 1.48)		77/64	0.83 (0.48, 1.44)	
Q4	148/69	0.84 (0.53, 1.33)		68/72	0.68 (0.39, 1.20)	

‡ *See Table 3 for the covariates*.

* *p < 0.05*,

** *p < 0.01*.

## Discussion Main Findings

We assessed the associations between six main one-carbon metabolism-related nutrients and fluorosis, and found that the dietary intakes of betaine, choline, methionine, folate, and vitamin B_6_ were inversely associated with fluorosis. These inverse associations were also observed in the choline compounds, such as phosphatidylcholine, free choline, glycerophosphocholine, and phosphocholine.

### Betaine, Choline, Methionine, and Fluorosis

Choline, betaine, and methionine have been identified as a key role in the methionine cycle, as they are essential nutrients for DNA synthesis and production of SAM (28). Many studies showed that an adequate intake of betaine and choline in the diet reduced the risk of disease (26, 29). In the present study, we first found protective associations of betaine, choline, and methionine with the occurrence of fluorosis. The previous studies have reported that a long-term methyl-deficient diet disrupted the DNA methylation *via* causing specific CpG hyper- or hypomethylation of the gene promoter regions (30). While methyl donors supplementation rectified the aberrant DNA methylation status. Our results indicated that sufficient betaine, choline, and methionine may act as methyl donors for epigenetic modification to prevent fluorosis (31). Recently, whole genome bisulfite sequencing and functional annotation of differentially methylated genes indicated that the alterations in methylation status of genes involved in biological processes are associated with bone development pathways. This study implied that fluoride induced DNA methylation of *BMP1, METAP2, MMP11*, and *BACH1* genes may hamper the extracellular matrix deposition, cartilage formation, angiogenesis, vascular system development, and porosity of bone (6). A previous study also revealed that fluoride had a dose-response effect on bone morphogenetic proteins in fluorosis rats, and fluoride induced hypomethylation of specific CpGs (12). Zhao et al. (15) reported that choline inhibited fluoride toxicity in the chondrocyte of rats and reduced the amount of fluoride in the organ tissues. In this regard, choline may play a methyl donor role against bone damage caused by excessive amounts of fluorine. Additionally, the associations between methionine intake and fluorosis were supported strongly by animal experiments due to methionine-supply fluorosis rats reduced lipid peroxidation in the liver (16), and enhanced antioxidant enzymes activity in the kidney (32). It was reported that betaine intake alleviated other chemical elements exposure induced impairments in rats, such as cadmium toxicity (33), arsenic poisoning (34), or the toxicity of paraquat (35). Consistent with these evidence, we observed that higher betaine intake was associated with a lower occurrence of fluorosis in the present study. However, the mechanisms of DNA methylation underlying these associations have not fully been defined yet.

### Folate, Vitamin B_6_, and Fluorosis

One-carbon metabolism is driven by the folate and requires B_6_ as a cofactor. Folate and B_6_ together regulate the DNA synthesis and methylation reactions (28). They play an important role in maintaining the stability of methylation patterns (28). We found that folate and vitamin B_6_ intakes were inversely associated with fluorosis. One previous cross-sectional study with 289 villagers showed that the dietary folate was a protective factor for skeletal fluorosis, with the adjusted *OR* (95% *CI*) of 0.76 (0.62–0.92) for folate intake (17). Our results were consistent with the above cross-sectional study. However, Pan et al. (18) observed that the plasma levels of homocysteine, vitamin B_6_, vitamin B_12_, and folate were not significantly different between 45 cases and 41 controls. Possible explanations for the inconsistencies were that plasma levels of nutrients reflected recent dietary intake rather than the long-term dietary intake and the small sample size may lead to false negative results. An epidemiologic study suggested that the methylation of the DNA repair gene O6-methylguanine transferase (*MGMT*) and the DNA mismatch repair gene *MLH1* in the blood of patients with coal-burning fluorosis were increased, and the methylation levels positively associated with the severity of fluorosis (36). The inverse association between dietary folate, vitamin B_6_ intake, and fluorosis were in accordance with the evidence that suggested high levels of folate and one-carbon metabolism-related B-vitamins can reduce the *MGMT* gene methylation (37).

### Vitamin B_12_, Sphingomyelin, and Fluorosis

Vitamin B_12_ functions as an enzyme that converts homocysteine to methionine and plays a role as a co-factor for methionine synthase (38). However, we reported no significant associations between dietary vitamin B_12_ and fluorosis. In the present study, the median intake of vitamin B_12_ was 2.21 μg per day, a relatively lower level compared with other one-carbon metabolism-related nutrients. Thus, it is hard to measure vitamin B_12_ accurately, which may lead to greater variation and weaken these associations (39). Consistent with this reasoning, the present study found no significant inverse association between dietary choline from sphingomyelin and fluorosis in our study population. This may be due to sphingomyelin being the least intake of all choline-containing compounds, with a median intake of 4.99 mg/day, while intake of the other four compounds ranged from 19.90 to 115.72 mg/day (non-fluorosis participants).

### Potential Biological Mechanism

The important role of methyl donors involved in DNA methylation should be taken into consideration due to aberrant DNA methylation was pathogenesis of fluorosis. For example, it was reported that IGF2 showed hypermethylation in the NaF-treated groups compared with the control group (40). However, dietary folate supplementation was demonstrated to negate IGF2 DNA hypermethylation induced by exposure to environmental pollutant (41), which was consistent with the upregulation of IGF2 expression. Herein, it was indicated that the dietary methyl donors may prevent the alterations of DNA methylation induced by fluoride. However, there were multiple other biologically plausible mechanisms for the beneficial effect of methyl donors on fluorosis. Oxidative damage has been known as one of the major mechanisms of fluorosis (42). Dietary folate, choline, and vitamin B_6_ may influence the fluorosis by means of their antioxidant properties, as they were well-documented antioxidant compounds that reduce the risk of diseases (43–45). Additionally, methionine supplementation may reduce the adverse effect of fluorosis on soft tissue *via* antioxidation (46). Therefore, the protective effect of one-carbon nutrients against fluorosis presented here may be reasonable. However, further studies are warranted to elucidate the pathophysiological mechanisms.

## Limitations

The potential limitations of this investigation should also cautiously be considered. First, a cross-sectional study design is unlikely to identify the causal relationship between one-carbon metabolism-related nutrients, although we minimized the potential reverse causation by excluding participants with other diseases, due to the changeable dietary habits of patients. Second, we could not preclude the possibility of recall bias. The consultation period for the dietary intake investigation was the year prior to the interview, and although we focused on the unbiased investigative techniques and fully documented the objective data, discrepancies could exist between the questionnaire and interview information. Third, the diagnosed fluorosis patients might change their lifestyle and dietary habits. However, public awareness of the fluorosis-preventing the effects of one-carbon metabolism-related nutrients was generally low, and thus the dietary evaluated information was unlikely misclassification between fluorosis and non-fluorosis participants. Furthermore, the consistency of the results across four compounds (choline from phosphatidylcholine, glycerophosphocholine, phosphocholine, and free choline) argues against bias to a certain extent, as these compounds are derived from different types of foods. Fourth, the use of USDA to calculate choline and betaine intakes was likely to increase the measurement errors in diet assessment. However, a previous study showed the validity of using the USDA database in the Chinese population to estimate choline and betaine intakes. Thus, the errors should be non-differential. Fifth, we did not exclude the vitamin intake from supplements, but sensitivity analysis by excluding participants with multi-vitamins supplements showed consistency with the primary outcomes. Finally, although we have adjusted for several confounding factors, some residual confounding may result from misclassification of those variables and confounding by unmeasured variables.

## Conclusions

In conclusion, dietary intakes of one-carbon metabolism-related nutrients betaine, choline, methionine, folate, and vitamin B_6_ were inversely associated with fluorosis occurrence. These results should encourage further studies on dietary one-carbon metabolism-related nutrients and fluorosis prevention to elucidate any measure applicability for dietary intervention.

## Data Availability Statement

The original contributions presented in the study are included in the article/Supplementary Material, further inquiries can be directed to the corresponding authors.

## Ethics Statement

The studies involving human participants were reviewed and approved by the Ethical Committee of Zunyi medical university (No.2014-1-1003). All participants in this study provided written informed consent before the interview. The patients/participants provided their written informed consent to participate in this study.

## Author Contributions

TC analyzed the data and wrote the manuscript. NT revised the manuscript and reply to comments of the reviewer, and also carried out the study of survey and determination. SY analyzed the data. DC carried out the study of survey and determination. XZ was responsible for organization and survey. DW and JL conceived and designed the study and reviewed the manuscript. All authors participated in data interpretation, reviewed, and approved the final draft.

## Funding

This study was supported by the National Natural Science Foundation of China (grant number 81460497; 82060595) and Guizhou Province Foundation for postgraduate Scientific Research Fund Project YJSCXJH (2019)093.

## Conflict of Interest

The authors declare that the research was conducted in the absence of any commercial or financial relationships that could be construed as a potential conflict of interest.

## Publisher's Note

All claims expressed in this article are solely those of the authors and do not necessarily represent those of their affiliated organizations, or those of the publisher, the editors and the reviewers. Any product that may be evaluated in this article, or claim that may be made by its manufacturer, is not guaranteed or endorsed by the publisher.

## References

[1] Luo KL Li L Zhang SX. Coal-burning roasted corn and chili as the cause of dental fluorosis for children in southwestern China. J Hazard Mater. (2011) 185:1340–7. 10.1016/j.jhazmat.2010.10.052 21074315

[2] Baily K Chilton J Dahi E Dahi E Fewtrell L Magara Y. Fluoride in Drinking Water. WHO press (2006). p. 32–4.

[3] National Health Commission of the People's Republic of China. China Health Statistics Yearbook. Bejing: China union Medical college press (2018). p. 276.

[4] Wang Q Cui KP Xu YY Gao YL Zhao J Li DS. Coal-burning endemic fluorosis is associated with reduced activity in antioxidative enzymes and Cu/Zn-SOD gene expression. Environ Geochem Health. (2014) 36:107–15. 10.1007/s10653-013-9522-2 23567976

[5] Suzuki M Bandoski C Bartlett JD. Fluoride induces oxidative damage and SIRT1/autophagy through ROS-mediated JNK signaling. Free Radic Biol Med. (2015) 89:369–78. 10.1016/j.freeradbiomed.2015.08.015 26431905 PMC4684823

[6] Daiwile AP Tarale P Sivanesan S Naoghare PK Bafana A Parmar D. Role of fluoride induced epigenetic alterations in the development of skeletal fluorosis. Ecotoxicol Environ Saf. (2019) 169: 410–7. 10.1016/j.ecoenv.2018.11.035 30469026

[7] Chen T Liu J. Advances in epigenetic pathogenesis of fluorosis. Chinese J Endemiol. (2020) 39:698–702. 10.1111/jcmm.14185 30784186 PMC6433665

[8] Zhao L Zhang S An X Tan W Tang B Zhang X. Sodium fluoride affects DNA methylation of imprinted genes in mouse early embryos. Cytogenet Genome Res. (2015) 147:41–7. 10.1159/000442067 26633825

[9] Zhu JQ Si YJ Cheng LY Xu BZ Wang QW Zhang X. Sodium fluoride disrupts DNA methylation of H19 and Peg3 imprinted genes during the early development of mouse embryo. Arch Toxicol. (2014) 88:241–8. 10.1007/s00204-013-1122-5 24030355 PMC3906544

[10] Pan X Yan W Qiu B Liao Y Liao Y Wu S. Aberrant DNA methylation of Cyclind-CDK4-p21 is associated with chronic fluoride poisoning. Chem Biol Interact. (2020) 315:108875. 10.1016/j.cbi.2019.108875 31669217

[11] Wang A Ma Q Gong B Sun L Afrim FK Sun R. DNA methylation and fluoride exposure in school-age children: epigenome-wide screening and population-based validation. Ecotoxicol Environ Saf . (2021) 223:112612. 10.1016/j.ecoenv.2021.112612 34371455

[12] Ma Y Yao Y Zhong N Angwa LM Pei J. The dose-time effects of fluoride on the expression and DNA methylation level of the promoter region of BMP-2 and BMP-7 in rats. Environ Toxicol Pharmacol. (2020) 75:103331. 10.1016/j.etap.2020.103331 32004919

[13] Zhang CX Pan MX Li B Wang L Mo XF Chen YM. Choline and betaine intake is inversely associated with breast cancer risk: a two-stage case-control study in China. Cancer Sci. (2013) 104:250–8. 10.1111/cas.12064 23140534 PMC7657205

[14] Xu X Chen J. One-carbon metabolism and breast cancer: an epidemiological perspective. J Genet Genomics. (2009) 36:203–14. 10.1016/S1673-8527(08)60108-3 19376481 PMC2694962

[15] Zhao Y Hao J Wang J Wang J. Effect of choline on the composition and degradation enzyme of extracellular matrix of mice chondrocytes exposed to fluoride. Biol Trace Elem Res. (2017) 175:414–20. 10.1007/s12011-016-0787-z 27368532

[16] Błaszczyk I Grucka-Mamczar E Kasperczyk S Birkner E. Influence of methionine upon the concentration of malondialdehyde in the tissues and blood of rats exposed to sodium fluoride. Biol Trace Elem Res. (2009) 129:229–38. 10.1007/s12011-008-8308-3 19137267

[17] Liu G Ye Q Chen W Zhao ZJ Li L Lin P. Study of the relationship between the lifestyle of residents residing in fluorosis endemic areas and adult skeletal fluorosis. Environ Toxicol Pharmacol. (2015) 40:326–32. 10.1016/j.etap.2015.06.022 26183810

[18] Pan J Wu CX Li Y Zhang C Chen T G ZZ. Changes in human blood H2S, Hcy, VitB6, VitB_12_ and folic acid levels in coal-burning endemic fluorosis areas. Chinese Pharmacol Bull. (2014) 30:250–2. 10.3969/j.issn.1001-1978.2014.02.023

[19] Perrier F Viallon V Ambatipudi S Ghantous A Cuenin C Hernandez-Vargas H. Association of leukocyte DNA methylation changes with dietary folate and alcohol intake in the EPIC study. Clin Epigenetics. (2019) 11:57. 10.1186/s13148-019-0637-x 30940212 PMC6444439

[20] Liu J Yang S Luo MJ Chen T Ma XJ Tao N. Association between dietary patterns and fluorosis in Guizhou, China. Front Nutr. (2019) 6:189. 10.3389/fnut.2019.00189 32039225 PMC6985547

[21] Liu J Yang S Luo MJ Zhao X Zhang YM Luo Y. Association of dietary carotenoids intake with skeletal fluorosis in the coal-burning fluorosis area of Guizhou Province. Biomed Environ Sci. (2018) 31:438–47. 30025556 10.3967/bes2018.057

[22] Ministry of Health of the People's Republic of China. The Normal Concentration of Urinary Fluoride of Population (WS/T 256–2005) 2005.

[23] Zhang CX Ho SC. Validity and reproducibility of a food frequency Questionnaire among Chinese women in Guangdong province. Asia Pac J clin Nutr. (2009) 18:240–50. 19713184

[24] Yang YX Wang GY Pan XC. China Food Composition, 2nd Edn. Beijin: University Medical Publishing House (2009).

[25] USDA national nutrient database for standard reference. Release 28. Available online at: http://www.ars.usda.gov/Services/docs.htm?docid=8964. (accessed Aug 20, 2016).

[26] Yang JJ Lipworth LP Shu XO Blot WJ Xiang YB Steinwandel MD. Associations of choline-related nutrients with cardiometabolic and all-cause mortality: results from 3 prospective cohort studies of blacks, whites, and Chinese. Am J Clin Nutr. (2020) 111:644–56. 10.1093/ajcn/nqz318 31915809 PMC7049525

[27] Willett W Stampfer MJ. Total energy intake: implications for epidemiologic analyses. Am J Epidemiol. (1986) 124:17–27. 10.1093/oxfordjournals.aje.a114366 3521261

[28] Lyon P Strippoli V Fang B Cimmino L. B Vitamins and one-carbon metabolism: implications in human health and disease. Nutrients. (2020) 12:2867. 10.3390/nu12092867 32961717 PMC7551072

[29] Łoboś P Regulska-Ilow B. Link between methyl nutrients and the DNA methylation process in the course of selected diseases in adults. Rocz Panstw Zakl Hig. (2021)72:123–36. 10.32394/rpzh.2021.0157 34114759

[30] Tryndyak VP Han T Muskhelishvili L Fuscoe JC Ross SA Beland FA. Coupling global methylation and gene expression profiles reveal key pathophysiological events in liver injury induced by a methyl-deficient diet. Mol Nutr Food Res. (2011) 55:411–8. 10.1002/mnfr.201000300 20938992

[31] Wang LJ Zhang HW Zhou JY Liu Y Yang Y Chen XL. Betaine attenuates hepatic steatosis by reducing methylation of the MTTP promoter and elevating genomic methylation in mice fed a high-fat diet. J Nutr Biochem. (2014) 25:329–36. 10.1016/j.jnutbio.2013.11.007 24456734

[32] Błaszczyk I Grucka-Mamczar E Kasperczyk S Birkner E. Influence of methionine upon the activity of antioxidative enzymes in the kidney of rats exposed to sodium fluoride. Biol Trace Elem Res. (2010) 133:60–70. 10.1007/s12011-009-8412-z 19488683

[33] Hagar H Al Malki W. Betaine supplementation protects against renal injury induced by cadmium intoxication in rats: role of oxidative stress and caspase-3. Environ Toxicol Pharmacol. (2014) 37:803–11. 10.1016/j.etap.2014.02.013 24632105

[34] Tazari M Baghshani H Moosavi Z. Effect of betaine versus arsenite-induced alterations of testicular oxidative stress and circulating androgenic indices in rats. Andrologia. (2018) 50:e13091. 10.1111/and.13091 29987898

[35] Na JD Choi YJ Jun DS Kim YC. Alleviation of paraquat-induced oxidative lung injury by betaine via regulation of sulfur-containing amino acid metabolism despite the lack of betaine-homocysteine methyltransferase (BHMT) in the lung. Food Funct. (2019) 10:1225–34. 10.1039/C8FO01457D 30746538

[36] Wu CX Wang YH Li Y Guan ZZ Qi XL. Changes of DNA repair gene methylation in blood of chronic fluorosis patients and rats. J Trace Elem Med Biol. (2018) 50:223–8. 10.1016/j.jtemb.2018.07.010 30262283

[37] Yang Y. The Role of One-Carbon Metabolism-Related Vitamins and MGMT Gene Methylation in the Development of Esophageal Cancer [D]. Southeast University (2015).

[38] Huang JY Butler LM Wang R Jin A Koh WP Yuan JM. Dietary intake of one-carbon metabolism-related nutrients and pancreatic cancer risk: the Singapore Chinese Health Study. Cancer Epidemiol Biomarkers Prev. (2016)25:417–24. 10.1158/1055-9965.EPI-15-0594 26711329 PMC4767683

[39] Beaton GH. Approaches to analysis of dietary data: relationship between planned analyses and choice of methodology. Am J Clin Nutr. (1994) 59:253s–61s. 10.1093/ajcn/59.1.253S 8279436

[40] Fu M Wu X He J Zhang Y Hua S. Natrium fluoride influences methylation modifications and induces apoptosis in mouse early embryos. Environ Sci Technol. (2014) 48:10398–405. 10.1021/es503026e 25102367

[41] Mao Z Xia W Huo W Zheng T Bassig BA Chang H. Pancreatic impairment and Igf2 hypermethylation induced by developmental exposure to bisphenol A can be counteracted by maternal folate supplementation. J Appl Toxicol. (2017) 37:825–35. 10.1002/jat.3430 28165156

[42] Varol E Icli A Aksoy F Bas HA Sutcu R Ersoy IH. Evaluation of total oxidative status and total antioxidant capacity in patients with endemic fluorosis. Toxicol Ind Health. (2013) 29:175–80. 10.1177/0748233711428641 22155887

[43] Zhang Y Kato H Sato H Yamaza H Hirofuji Y Han X. Folic acid-mediated mitochondrial activation for protection against oxidative stress in human dental pulp stem cells derived from deciduous teeth. Biochem Biophys Res Commun. (2019) 508:850–6. 10.1016/j.bbrc.2018.11.169 30528238

[44] Yang M Kuang M Wang G Ali I Tang Y Yang C. Choline attenuates heat stress-induced oxidative injury and apoptosis in bovine mammary epithelial cells by modulating PERK/Nrf-2 signaling pathway. Mol Immunol. (2021) 135:388–97. 10.1016/j.molimm.2021.05.002 34022514

[45] Matxain JM Padro D Ristila M Strid A Eriksson LA. Evidence of high ^*^OH radical quenching efficiency by vitamin B6. J Phys Chem B. (2009) 113:9629–32. 10.1021/jp903023c 19558175

[46] Błaszczyk I Birkner E Gutowska I Romuk E Chlubek D. Influence of methionine and vitamin E on fluoride concentration in bones and teeth of rats exposed to sodium fluoride in drinking water. Biol Trace Elem Res. (2012) 146:335–9. 10.1007/s12011-011-9251-2 22068731

