# Supplementary material for: Association Between Dietary Intake of One-Carbon Metabolism-Related Nutrients and Fluorosis in Guizhou, China
Source: Front Nutr. 2021 Sep 28;8:700726. doi: 10.3389/fnut.2021.700726 (PMC8505735; doi:10.3389/fnut.2021.700726)
Supplement: Supplementary file 2 [file Data_Sheet_1.pdf]

Table S1. Association between Quartiles of Dietary Energy-adjusted Folate and Fluorosis According to Sex, Alcohol Drinking Status, Smoking Status and Urinary Fluoride Levels

Folate (ug/day)	Quaritle of intake				P-trend	P-interaction
	Q1	Q2	Q3	Q4		
Sex						
Men						
No. cases/controls	67/12	79/14	83/36	82/39		
OR (95% CI) ‡	1	1.09 (0.45,2.67)	0.36 (0.16,0.80)	0.37 (0.17,0.83)	0.001	
Women						
No. cases/controls	112/32	98/33	70/35	62/40		
OR (95% CI) ‡	1	0.84 (0.46,1.54)	0.52 (0.28,0.98)	0.41(0.21, 0.79)	0.003	0.960
Alcohol						
Drinker						
No. cases/controls	40/12	64/11	46/24	52/26		
OR (95% CI) ‡	1	1.94 (0.70,5.46)	0.51 (0.20,1.35)	0.65 (0.26,1.63)	0.063	
Non-drinker						
No. cases/controls	139/32	113/36	107/47	92/53		
OR (95% CI) ‡	1	0.67(0.38,1.18)	0.43 (0.24,0.76)	0.30 (0.17, 0.56)	< 0.001	0.591
Smoking status						
Smoker						
No. cases/controls	64/15	70/12	72/28	71/30		
OR (95% CI) ‡	1	1.50 (0.61,3.69)	0.55 (0.25,1.22)	0.55 (0.25,1.21)	0.032	
Non-smoker						

No. cases/controls	115/29	107/35	81/43	73/49		
OR (95% CI) [‡]	1	0.73(0.40,1.33)	0.41 (0.22,0.77)	0.33 (0.17, 0.62)	< 0.001	0.290
Urinary level [†]						
≤1.6 mg/L						
No. cases/controls	84/25	88/20	74/36	78/50		
OR (95% CI) [‡]	1	1.19 (0.57,2.46)	0.51 (0.26,1.01)	0.31 (0.16,0.61)	< 0.001	
>1.6mg/L						
No. cases/controls	56/10	58/11	56/14	46/12		
	1	0.94 (0.34,2.64)	0.47 (0.17,1.34)	0.60(0.21, 1.76)	0.214	0.584

Note. [‡] odds ratio was adjusted for covariates including age, gender, marital status, education level, income, smoking status, passive smoking status, alcohol drinking status, tea drinking status , using coal to roast grains or chili, washing dry grains or chili before use, fuel type, using improved stove, calcium intake, roasted chili and grains consumption, total energy intake. [†] We obtained 718 urinary fluoride measurements from participants. [*] $P < 0.05$, [**] $P < 0.01$.

Table S2. Association between Quartiles of Dietary Energy-adjusted Methionine and Fluorosis According to Sex, Alcohol Drinking Status, Smoking Status and Urinary Fluoride Levels

Methionine (mg/day)	Quaritle of intake				P-trend	P-interaction
	Q1	Q2	Q3	Q4		
Sex						
Men						
No. cases/controls	66/13	80/16	81/25	84/47		
OR (95% CI) ‡	1	1.18 (0.50,2.77)	0.67 (0.29,1.54)	0.37 (0.17,0.81)	0.001	
Women						
No. cases/controls	105/39	100/28	83/35	54/38		
OR (95% CI) ‡	1	1.09 (0.58,2.02)	0.75 (0.41,1.38)	0.51(0.27, 0.96)	0.024	0.603
Alcohol						
Drinker						
No. cases/controls	43/11	56/15	53/14	50/33		
OR (95% CI) ‡	1	1.11 (0.41,0.30)	1.24 (0.44,3.50)	0.44 (0.17,1.15)	0.040	
Non-drinker						
No. cases/controls	128/41	124/29	111/46	88/52		
OR (95% CI) ‡	1	1.16 (0.65,2.06)	0.64 (0.37,1.10)	0.47 (0.27,0.83)	0.002	0.893
Smoking status						
Smoker						
No. cases/controls	64/16	73/14	72/19	68/36		
OR (95% CI) ‡	1	1.49 (0.64,3.51)	1.00 (0.43,2.36)	0.49 (0.22,1.07)	0.016	
Non-smoker						
No. cases/controls	107/36	107/30	92/41	70/49		

OR (95% CI) [‡]	1	1.04(0.57,1.91)	0.64 (0.36,1.16)	0.47 (0.25,0.85)	0.005	0.734
Urinary level [†]						
≤1.6 mg/L						
No. cases/controls	74/25	91/20	82/29	77/57		
OR (95% CI) [‡]	1	1.45 (0.67,3.06)	0.81 (0.40,1.65)	0.31 (0.16,0.62) [**]	< 0.001	
>1.6mg/L						
No. cases/controls	58/15	62/8	56/15	40/9		
	1	1.87 (0.68,5.14)	1.01 (0.41,2.51)	1.28 (0.43, 3.75)	0.888	0.147

Note. [‡] See table S1 for the covariates. [†] We obtained 718 urinary fluoride measurements from participants. [*] $P < 0.05$, [**] $P < 0.01$.

Table S3. Association between Quartiles of Dietary Energy-adjusted Vitamin B_6 and Fluorosis According to Sex, Alcohol Drinking Status, Smoking Status and Urinary Fluoride Levels

Vitamin B_6 (mg/day)	Quaritle of intake				P-trend	P-interaction
	Q1	Q2	Q3	Q4		
Sex						
Men						
No. cases/controls	71/10	76/17	86/38	78/36		
OR (95% CI) ‡	1	0.60 (0.25,1.47)	0.26 (0.11,0.60) **	0.33 (0.14,0.76) **	0.003	
Women						
No. cases/controls	106/36	104/27	67/33	65/44		
OR (95% CI) ‡	1	1.24(0.67,2.32)	0.70 (0.37,1.32)	0.36 (0.19,0.69) **	0.001	0.877
Alcohol						
Drinker						
No. cases/controls	42/7	64/16	45/24	51/26		
OR (95% CI) ‡	1	0.56 (0.19,1.62)	0.27 (0.09,0.79) *	0.31 (0.11,0.88) *	0.015	
Non-drinker						
No. cases/controls	135/39	116/28	108/47	92/54		
OR (95% CI) ‡	1	1.12 (0.62,2.00)	0.53 (0.30,0.92) *	0.37 (0.21,0.66) **	< 0.001	0.862
Smoking status						
Smoker						
No. cases/controls	67/12	74/18	72/29	64/26		
OR (95% CI) ‡	1	0.79 (0.33,1.89)	0.34 (0.15,0.79) *	0.50 (0.21,1.19)	0.041	
Non-smoker						
No. cases/controls	110/34	106/26	81/42	79/54		

OR (95% CI) ‡	1	1.11 (0.59,2.08)	0.53 (0.31,1.05)	0.32 (0.17,0.59) **	< 0.001	0.655
Urinary level †						
≤1.6 mg/L						
No. cases/controls	88/26	85/20	72/34	79/51		
OR (95% CI) ‡	1	1.12 (0.55,2.31)	0.49 (0.24,0.98) *	0.31 (0.16,0.60) **	< 0.001	
>1.6mg/L						
No. cases/controls	52/12	65/10	54/16	45/9		
	1	1.43 (0.52,3.94)	0.65 (0.24,1.75)	0.99 (0.33,3.01)	0.628	0.273

Note. ‡ See table S1 for the covariates. † We obtained 718 urinary fluoride measurements from participants. * $P< 0.05$, ** $P< 0.01$.

Table S4. Association between Quartiles of Dietary Energy-adjusted Total Choline and Fluorosis According to Sex, Alcohol Drinking Status, Smoking Status and Urinary Fluoride Levels

Total choline (mg/day)	Quaritle of intake				P-trend	P-interaction
	Q1	Q2	Q3	Q4		
Sex						
Men						
No. cases/controls	77/11	71/21	77/35	86/34		
OR (95% CI) ‡	1	0.49 (0.21,1.13)	0.32 (0.14,0.71) **	0.43 (0.19,0.97) *	0.044	
Women						
No. cases/controls	103/32	97/35	83/29	59/44		
OR (95% CI) ‡	1	0.73 (0.39,1.35)	0.94 (0.49,1.79)	0.39 (0.20,0.74) **	0.016	0.662
Alcohol						
Drinker						
No. cases/controls	44/11	48/13	53/26	57/23		
OR (95% CI) ‡	1	1.02 (0.38,2.76)	0.32 (0.25,1.46)	0.85 (0.34,2.12)	0.519	
Non-drinker						
No. cases/controls	136/32	120/43	107/38	88/55		
OR (95% CI) ‡	1	0.53 (0.30,0.94) *	0.59 (0.33,1.06)	0.35 (0.20,0.63) **	0.001	0.368
Smoking status						
Smoker						
No. cases/controls	66/12	68/22	73/26	70/25		
OR (95% CI) ‡	1	0.58 (0.25,1.32)	0.55 (0.24,1.25)	0.69 (0.30,1.60)	0.480	
Non-smoker						
No. cases/controls	114/31	100/34	87/38	75/53		

OR (95% CI) ‡	1	0.68 (0.37,1.25)	0.61 (0.33,1.12)	0.35 (0.19,0.64) **	0.001	0.093
Urinary level †						
≤1.6 mg/L						
No. cases/controls	76/20	82/34	89/29	77/48		
OR (95% CI) ‡	1	0.53 (0.26,1.07)	0.65 (0.31,1.35)	0.35 (0.17,0.70) **	0.008	
>1.6mg/L						
No. cases/controls	64/12	63/9	42/16	47/10		
	1	1.48 (0.50,4.37)	0.58 (0.22,1.55)	1.20 (0.41,3.49)	0.749	0.498

Note. ‡ See table S1 for the covariates.† We obtained 718 urinary fluoride measurements from participants. * $P < 0.05$, ** $P < 0.01$.

Table S5. Association between Quartiles of Dietary Energy-adjusted Betaine and Fluorosis According to Sex, Alcohol Drinking Status, Smoking Status and Urinary Fluoride Levels

Betaine (mg/day)	Quaritle of intake				P-trend	P-interaction
	Q1	Q2	Q3	Q4		
Sex						
Men						
No. cases/controls	70/18	81/19	84/30	76/34		
OR (95% CI) ‡	1	1.27 (0.59,2.73)	0.68 (0.33,1.41)	0.72 (0.34,1.50) *	0.153	
Women						
No. cases/controls	103/32	93/31	78/32	68/45		
OR (95% CI) ‡	1	0.90 (0.48,1.69)	0.78 (0.41,1.48)	0.51 (0.27,0.95)	0.032	0.453
Alcohol						
Drinker						
No. cases/controls	47/12	48/16	55/15	52/30		
OR (95% CI) ‡	1	0.79 (0.31,2.03)	0.82 (0.31,2.14)	0.60 (0.25,1.43)	0.264	
Non-drinker						
No. cases/controls	126/38	126/34	107/47	92/49		
OR (95% CI) ‡	1	1.14 (0.65,1.99)	0.66 (0.38,1.14)	0.59 (0.33,1.04)	0.020	0.809
Smoking status						
Smoker						
No. cases/controls	65/17	76/18	66/24	70/26		
OR (95% CI) ‡	1	1.20 (0.54,2.66)	0.68 (0.32,1.48)	0.91 (0.42,1.96)	0.476	
Non-smoker						
No. cases/controls	108/33	98/32	96/38	74/53		

OR (95% CI) ‡	1	0.96 (0.52,1.77)	0.75 (0.41,1.39)	0.45 (0.25,0.82) **	0.006	0.149
Urinary level †						
≤1.6 mg/L						
No. cases/controls	70/23	95/22	83/34	76/52		
OR (95% CI) ‡	1	1.26 (0.61,2.58)	0.58 (0.29,1.16)	0.47 (0.24,0.91) *	0.004	
>1.6mg/L						
No. cases/controls	66/12	61/13	43/14	46/8		
	1	0.80 (0.30,2.14)	0.56 (0.21,1.50)	1.06 (0.35,3.20)	0.766	0.205

Note. ‡ See table S1 for the covariates. † We obtained 718 urinary fluoride measurements from participants. * P<0.05, ** P< 0.01.
